# Relationships between *Hyalesthes obsoletus*, Its Herbaceous Hosts and Bois Noir Epidemiology in Northern Italian Vineyards

**DOI:** 10.3390/insects11090606

**Published:** 2020-09-07

**Authors:** Nicola Mori, Elena Cargnus, Marta Martini, Francesco Pavan

**Affiliations:** 1 Department of Biotechnology, University of Verona, Strada Le Grazie 15, 37134 Verona, Italy; nicola.mori@univr.it; 2 Department of Agricultural, Food, Environmental and Animal Sciences, University of Udine, Via delle Scienze 206, 3100 Udine, Italy; elena.cargnus@uniud.it (E.C.); marta.martini@uniud.it (M.M.)

**Keywords:** Cixiidae, grapevine yellows diseases, “*Ca*. Phytoplasma solani”, *Convolvolus arvensis*, *Urtica dioica*

## Abstract

**Simple Summary:**

Bois noir is a phytoplasma disease causing heavy yield losses in European vineyards mainly transmitted by the planthopper *Hyalesthes obsoletus.* There are two main molecular types of the phytoplasma causal agent acquired from *Convolvolus arvensis* and *Urtica dioica*, respectively. Previous studies showed biological and genetic differences in the *H. obsoletus* populations associated with the two host plants and respective phytoplasma molecular types. Over a six-year study, the relationship between *H. obsoletus* phenology and its yearly abundance, and spatial distribution of both vector and its host plants was studied in northern Italian infected vineyards. The results showed clear differences in the two *H. obsoletus* populations (i.e., earlier phenology on *C. convolvulus*, adult better survival on the host plant where nymphs developed, adult distribution inside or outside vineyards according to *C. arvensis* and *U. dioica* presence) and supported the hypothesis of a cryptic speciation. Moreover, an influence of late frosts in spring on nymphal mortality was found. The differences in phenology and spatial distribution of the two *H. obsoletus* populations associated with the two herbaceous host plants have practical consequences in the Bois noir management.

**Abstract:**

*Hyalesthes obsoletus* is the vector of “*Candidatus* Phytoplasma (*Ca*. P.) solani,” the causal agent of grapevine yellows Bois noir (BN). The relationships among the planthopper, its main herbaceous hosts as phytoplasma reservoirs (*Convolvolus arvensis* and *Urtica dioica*) and BN spreading were studied in northern Italy. In two areas the relationship between host plants and the phenology and survival of planthopper adults was investigated in potted plants and in field conditions. Moreover, *H. obsoletus* ecology, newly symptomatic grapevine occurrence and “*Ca*. P. solani” *tuf*-types’ presence were studied in two vineyards (2014–2019). An earlier occurrence of *H. obsoletus* adults on *C. arvensis* than *U. dioica* and better adult survival of the originating host were observed. When *U. dioica* was prevalent, the vector occurred almost exclusively along the ditch outside the vineyard. *Hyalesthes obsoletus* amount varied widely from year to year and nymphal mortality due to late frosts was supposed. In one vineyard, the amount of newly symptomatic grapevines was significantly correlated with vector abundance in the previous year. The “*Ca*. P. solani” *tuf*-type was influenced by vector population levels on the two hosts. Since the abundance of *H. obsoletus* populations on the two hosts influences BN epidemiology and dynamics and the “*Ca*. P. solani” *tuf*-type, this must be considered in BN control strategies.

## 1. Introduction

Bois noir (BN) is the most widespread grapevine yellows disease (GY) in the Euro-Mediterranean, where it causes yield losses and grapevine death [1,2]. BN is associated with grapevine infections by “*Candidatus* Phytoplasma (*Ca*. P.) solani” (subgroup 16SrXII-A) [3,4]. The main “*Ca*. P. solani” vector is *Hyalesthes obsoletus* Signoret (Hemiptera: Cixiidae) [5,6,7,8,9,10,11,12], a polyphagous planthopper living preferentially on herbaceous plants, such as stinging nettle (*Urtica dioica* L.), field bindweed (*Convolvulus arvensis* L.), stinking hawk’s-beard (*Crepis foetida* L.) and *Artemisia* spp.; and shrub plants, such as *Vitex agnus-castus* [7,9,12,13,14,15,16,17,18,19,20,21]. The role of *H. absoletus* in the spread of BN in vineyards is due to the fact that adults can occasionally feed on grapevines [5,6]. Recently, *Reptalus panzeri* (Low) (Hemiptera: Cixiidae) has been reported as vector of “*Ca*. P. solani” in Serbian vineyards [21,22]; moreover, other vectors can be involved in the BN epidemiology [23,24,25]. The sequence analysis of *tufB* gene revealed that two main “*Ca*. P. solani” *tuf*-types are present on grapevines and alternative host plants, according to diverse ecological pathosystems: (i) stinging nettle—*H. obsoletus*—grapevine *tuf*-type a, and (ii) field bindweed—*H. obsoletus*—grapevine *tuf*-type b [15]. According to these associations, PCR-positive *H. obsoletus* from *C. arvensis* were almost always infected by *tuf*-type b, whereas *tuf*-type a was prevalent in those collected from *U. dioica* [17,26,27,28,29].

In summer *H. obsoletus* females lay eggs on the ground near the roots of their herbaceous hosts, and nymphs complete their development from late summer to the next spring [13]. However, on field bindweed and stinging nettle plants, the phenology of *H. obsoletus* is different. On *C. arvensis*, adult emergence begins earlier than on *U. dioica* in different European countries, such as in Italy [30,31], Germany [26,32] and Serbia [18]. In northern Italy *H. obsoletus* overwinters as third nymphal instars on field bindweed and second ones on stinging nettle [19], whereas in Germany on both host plants overwintering occurs as second-third instars [33]. Based on the different phenology of adult emergence on the two herbaceous plants, two different phenological models were proposed [32,34]. *Hyalesthes obsoletus* adult populations collected on field bindweed and stinging nettle showed a better survival and adaptation to the originating host [26,35,36,37]. The genetic differences between the populations associated with these two herbaceous plants suggested the occurrence of cryptic speciation [38,39]. 

In grape-growing areas of northern Italy where BN is spreading, the influences of field bindweed and stinging nettle on *H. obsoletus* phenology and BN epidemiology were investigated. In particular, the study was conducted (1) to compare the phenology of *H. obsoletus* adults on field bindweed and stinging nettle growing in vineyard habitats; (2) to compare the spatial distribution and phenology of *H. obsoletus* adults between inside and outside the vineyards in relation to the presence of the two herbaceous host plants; (3) to verify the survival and adaptation of *H. obsoletus* adults on a host plant other than that on which the nymphs developed; (4) to verify the existence of a relationship between the population levels of *H. obsoletus* and newly symptomatic grapevines; (5) to verify the relationship between *H. obsoletus* adult phenology and “*Ca*. P. solani” *tuf*-type in newly symptomatic grapevines.

## 2. Materials and Methods

### 2.1. The Influence of the Host Plant on Adult Phenology and Distribution

During 2007 in two grape-growing areas of the Emilia-Romagna region (i.e., Modena and Reggio-Emilia districts) the *H. obsoletus* adult populations on stinging nettle and field bindweed were monitored weekly from June to August using a sweep net and a pooter. In each area, four Lambrusco vineyards, two with the exclusive presence of stinging nettle (Bomborto locality, 44°43′42″ N, 11°02′430″ E, 26 m a.s.l. for Modena district; Massenzatico locality, 44°44′06″ N, 10°41′53″ E, 37 m a.s.l for Reggio-Emilia district) and two with the exclusive presence of field bindweed (Formigine locality 44°34′22″ N, 10°50′50″ E, 81 m a.s.l. for Modena district; Sesso locality, 44°44′21″ N, 10°36′59″ E, 34 m a.s.l for Reggio-Emilia district) were surveyed. In the vineyards with stinging nettle or field bindweed, “*Ca*. P. solani” *tuf*-type a and *tuf*-type b, respectively, had been previously detected [17]. In another vineyard of Modena area (Soliera locality, 44°44′19″ N, 10°55′27″ E, 28 m a.s.l.) the ground cover vegetation showed the contemporary presence of stinging nettle and field bindweed. In each vineyard, *H. obsoletus* adults were sampled on herbaceous vegetation both in vineyard inter-rows (inside) and in the surroundings (outside). At each sampling, C. *arvensis* or *U. dioica* plants were swept 60 times, both inside and outside the vineyards, collecting live specimens with the pooter every six sweeps.

### 2.2. Adults’ Survival and Adaptation on Different Development Host Plants

*Hyalesthes obsoletus* adults were collected in Veneto region on 14th July 2007 from stinging nettle plants (N. 250), growing along a ditch bordering a BN-infected Chardonnay vineyard (45°23′35″ N, 11°09′39″ E, 28 m a.s.l.), and from field bindweed plants (N. 250), growing in a BN-infected Chardonnay vineyard (45°20′14″ N, 11°13′30″ E, 22 m a.s.l.). Ten caged pots were prepared with stinging nettle and another ten with field bindweed. The adults collected on stinging nettle were released in equal numbers on five stinging nettle pots and five field bindweed pots in order to have 25 adults per potted plant. The same procedure was followed with the adults from field bindweed. Therefore, for each plant, half of the adults were reared on the plant where their young stages originated and half on the other host plant.

After 1, 3, 7, 14, 21 and 28 days the number of live adults was counted on each potted plant and the survival rate was calculated for each group. For each *H. obsoletus* origin (i.e., from field bindweed or stinging nettle), Kaplan–Meier analysis was used to estimate the survival curves on the two plants, and the comparison between two survival curves was made by the log-rank test.

### 2.3. Adult Populations and Bois Noir Incidence

The investigation was conducted from 2014 to 2019, in two Chardonnay vineyards located in Friuli Venezia Giulia region (Gorizia district, Cormons locality). Both vineyards (vineyards A and B) were trained with Guyot system (distances between/along rows of 3.5/1.0 and 2.6/0.8 m, respectively). Vineyard A (45°56′34″ N, 13°26′45″ E, 44 m a.s.l.) measured 80 by 95 m with the shorter side facing a ditch 20 m away. Vineyard B (45°56′44″ N, 13°26′57″ E, 45 m a.s.l.) measured 16 by 200 m with the longer side facing a ditch 7 m away. Stinging nettle plants were present only along the ditch bordering the vineyards, whereas field bindweed plants were spread mostly inside the vineyard. The vineyards were treated in late June–early July with insecticides against *Scaphoideus titanus* Ball (Hemiptera: Cicadellidae) (neonicotinoids in vineyard A and organophosphate in vineyard B) and a standard fungicide program was followed. In the years before the study (from 2007 for vineyard A and 2011 for vineyard B) a significant decreasing gradient of symptomatic grapevines from the border next to the ditch with abundant stinging nettle was observed in both vineyards and all grapevines were affected by “*Ca.* P. solani” *tuf*-type a (data not reported).

From 2014 to 2019, grapevines were surveyed in early September to detect plants showing GY symptoms for the first time (hereafter named newly symptomatic grapevines).

From 2014 to 2018, in the habitats of vineyards A and B *H. obsoletus* populations were monitored with yellow sticky traps replaced weekly from early June to late July. Seven traps were placed along the ditch (hereafter named outside). In correspondence to each trap along the ditch, series of four and three traps were installed in vineyard A (at the border and after 12, 32 and 72 m) and in vineyard B (at the border and after 5 m and 16 m), respectively (hereafter named inside). Yellow sticky traps were made with plastic yellow sheets (11 cm wide, 21 cm long and 0.2 cm thick) (Plastibor s.r.l., Ponte San Nicolò, Padova, Italy) and smeared, on both sides, for four fifths of their surface with glue (Temo-O-Cid, Kollant Srl, Vigonovo VE, Italy). The traps were fixed to a stake in a vertical position with their lower part just above the herbaceous vegetation.

Traps were transferred to the laboratory and *H. obsoletus* specimens were identified and counted under a dissection microscope [40,41].

For each vineyard and year, the numbers of *H. obsoletus* adults (captures per trap) outside and inside the vineyards were plotted over time. An ANOVA general linear model was performed considering as sources of variation year, periods (i.e., the 25 days from 1 June to 25 June and the 25 days from 26 June to 20 July), positions (i.e., outside or inside the vineyard), replicates (i.e., seven positions) and interactions. Data were log+1 transformed to satisfy assumptions of a normal distribution. Captures were divided in two periods of equal duration (1–25 June and 26 June–20 July) in accordance with the data reported in this study (see § 3.1. of Results section) and [31], which showed that captures in June are mainly associated with field bindweed and in July with stinging nettle. The captures inside the vineyard were grouped per each of seven series and the average number of captures per trap was calculated. To compare the captures in the different years, a Tukey’s post hoc test was used. The ANOVA was performed with Minitab Inc. Minitab^®^ 16.

For each vineyard, linear regressions between the *H. obsoletus* yearly captures inside the vineyard, and the percentage of grapevines showing GY symptoms for the first time in the same year and the next year were calculated.

### 2.4. Molecular Identification and Characterization of “Ca. P. solani” in Newly Symptomatic Grapevines

Every year, a large number of the newly symptomatic grapevines were submitted to molecular analyses as described below.

After total genomic DNA extraction following a modified Doyle and Doyle method [42], the presence of phytoplasmas in symptomatic plants was determined by an EvaGreen qPCR protocol using phytoplasma universal primers 16S(RT)F1/16S(RT)R1 (138 bps) [43]. One microliter of diluted DNA (20 ng μL^−1^) was used as a template in 15 μL-PCR reactions including 0.3 μM each primer, 1 × Sso Fast EvaGreen SuperMix (Bio-Rad Inc., Hercules, CA, USA) and sterile H_2_O. Diluted total genomic DNA of phytoplasma reference strains maintained in periwinkle was used as positive control in real-time PCRs. Cycling conditions in a 96-well Bio-Rad CFX96 RealTime PCR Detection System (Bio-Rad Inc., Hercules, CA, USA) were as follows: initial denaturation at 98 °C for 2 min; 55 cycles of 5 s at 98 °C; 5 s at 57 °C. A high-resolution melting (HRM) analysis (ramp from 65 °C to 95 °C with 0.2 °C increments and holding times of 10 s) was programmed at the end of the cycling reaction and data were processed using the Precision Melt Analysis Software 1.0 (Bio-Rad Laboratories) [44].

“*Ca*. P. solani” strains identified in symptomatic grapevines were typed by PCR/RFLP analyses based on *tuf* gene using primers TufAYf/TufAYr followed by digestion with *Hpa*II [15,45]. Amplifications were performed with the automated One Advanced thermocycler (EuroClone, Celbio, Milan, Italy) in 25 μL reactions containing 200 μM of each of the four dNTPs, 0.4 μM of each primer, 1.5 mM MgCl_2_, 0.625 units of GoTaq Flexi DNA Polymerase (Promega, Madison, WI, USA) and 1 μL of diluted DNA (20 ng μL^−1^). The PCR program consisted of initial denaturation for 2 min at 94 °C; 40 cycles of 1 min at 94 °C, 45 s at 53 °C and 1.5 min at 72 °C; and a final extension for 8 min at 72 °C.

To compare the proportions of plants infected with *tuf*-type a and *tuf*-type b, the binomial test of significance was used.

## 3. Results

### 3.1. Influence of Host Plant on the Adult Phenology and Distribution

**Figure 1:**
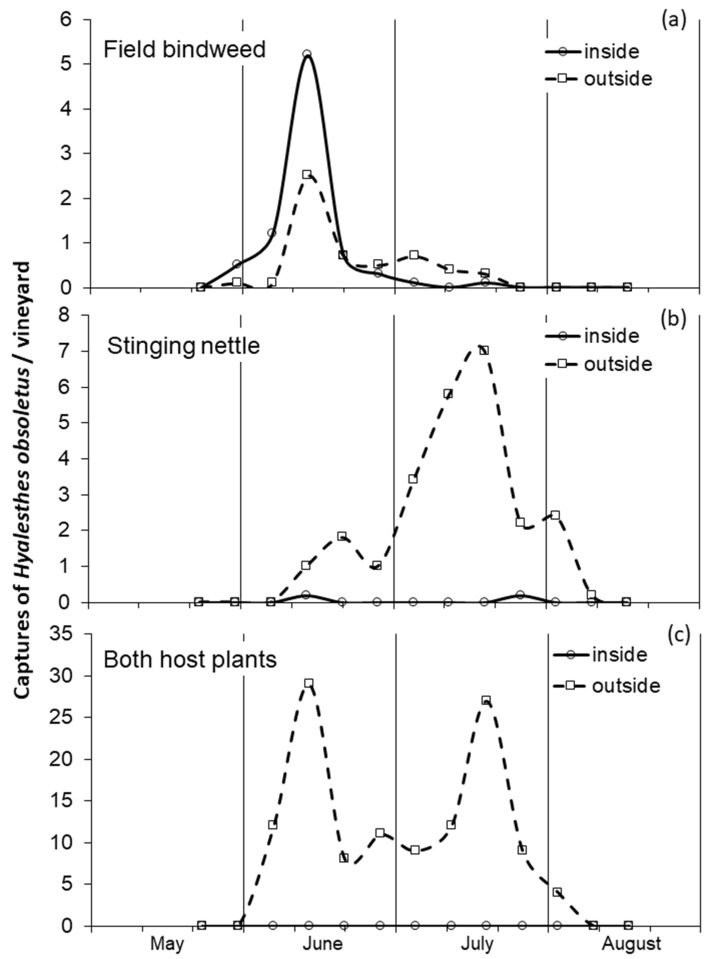
Dynamics of captures of *Hyalesthes obsoletus* recorded during 2007 in Emilia-Romagna region: (**a**) in two vineyards with the exclusive presence of field bindweed; (**b**) in two vineyards with the exclusive presence of stinging nettle; (**c**) in one vineyard with both host plants. Adults were sampled both inside and outside vineyards.

On field bindweed in both grape-growing areas of Emilia-Romagna region the adults of *H. obsoletus* were observed from late May to the second 10 days of July with a peak of captures in mid-June, and captures were abundant both inside and outside the vineyards (Figure 1a). On stinging nettles in both grape-growing areas, the adults of *H. obsoletus* were observed from mid-June to early-August with a peak of captures in mid-July, and were more abundant outside than inside according to a different presence of the host plant (Figure 1b). On stinging nettle in comparison with field bindweed, the beginning of captures was delayed by two weeks; the peak was even delayed by a month and the end by three weeks. In the vineyard with both stinging nettle and field bindweed, two distinct peaks of *H. obsoletus* captures were observed, coinciding with those recorded in the vineyards with field bindweed and in those with stinging nettle, respectively (Figure 1c).

### 3.2. Adults’ Survival and Adaptation on Different Development Host Plants

**Figure 2:**
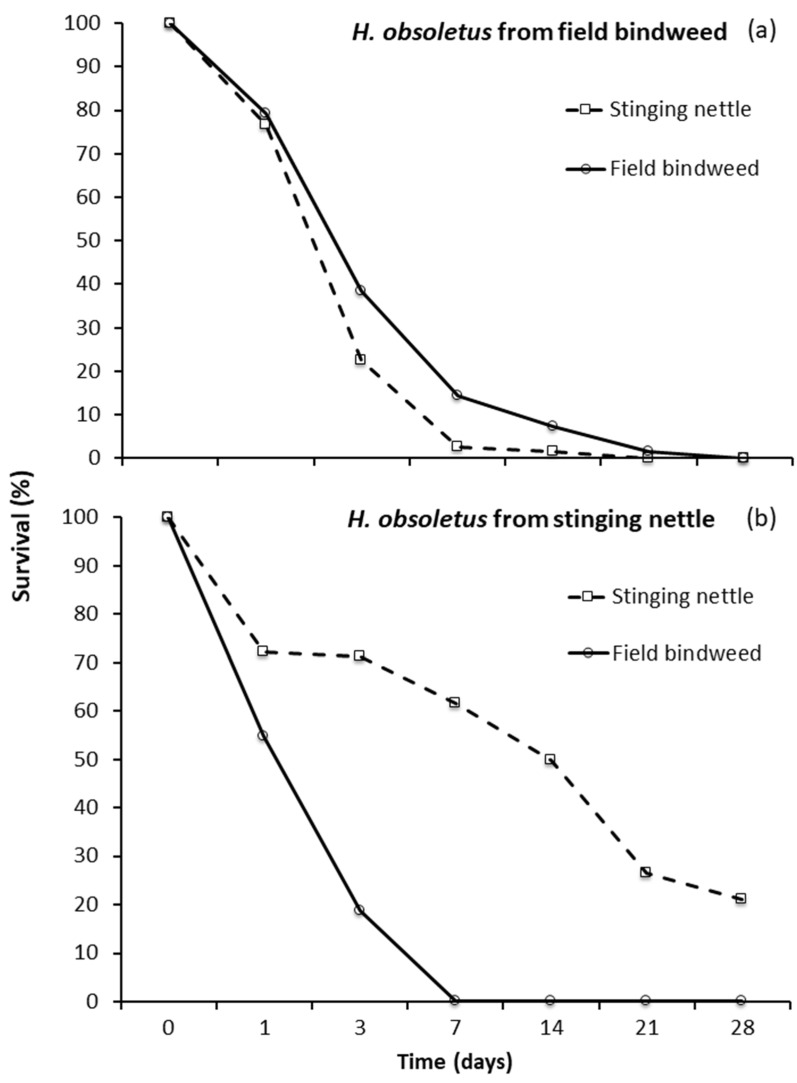
Survival in caged pot plants of *Hyalesthes obsoletus* adults: emerged (**a**) from field bindweed or (**b**) stinging nettle and released on both plants.

*Hyalesthes obsoletus* adults, released on the same plant species on which the nymphs had developed, survived longer than those released on the other host plant (Figure 2a,b) (from field bindweed: *Χ*^2^ = 38.96, *p* < 0.0001; from stinging nettle: *Χ*^2^ = 72.10, *p* < 0.0001). When adults were released on a host plant different from the one on which the nymphs had developed, they died in seven days.

### 3.3. Factors Influencing Adult Abundance and Phenology in Vineyard Habitats

In both vineyard habitats, the captures of *H. obsoletus* adults were significantly influenced by year and period (Table 1 and Table 2). In vineyard A habitat, the position also significantly influenced the captures.

**Figure 3:**
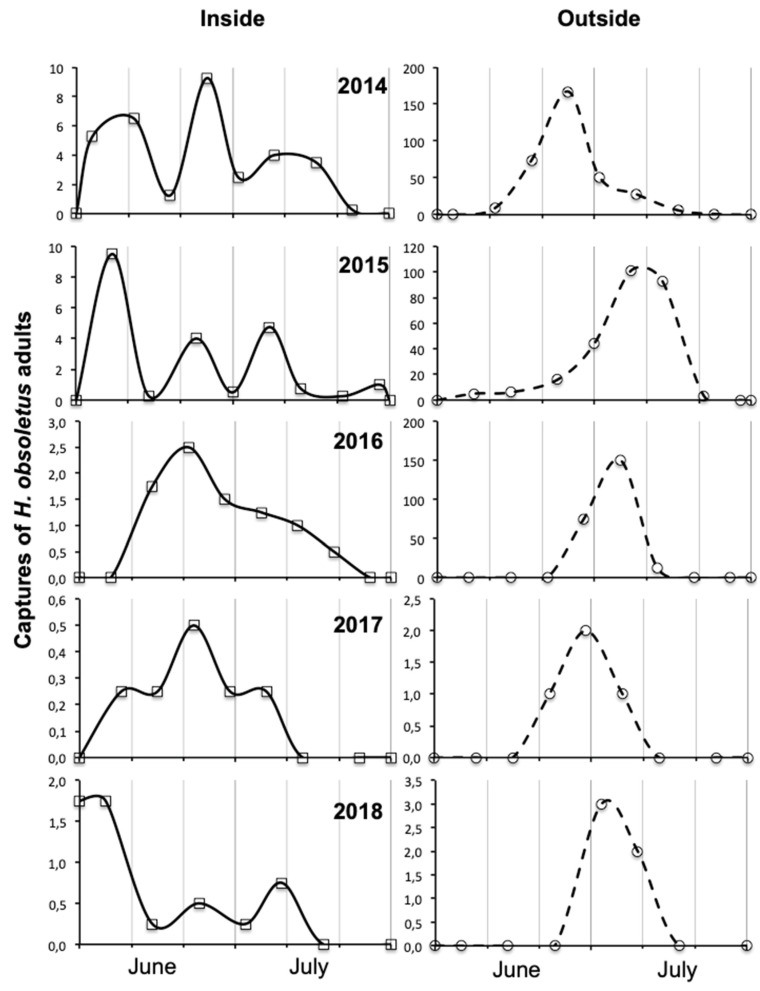
*Hyalesthes obsoletus* captured on yellow sticky traps during 2014–2018 both inside and outside vineyard A. A value of captures for outside refers to the sum of seven traps and for inside to the sum of the average of each of seven series.

**Figure 4:**
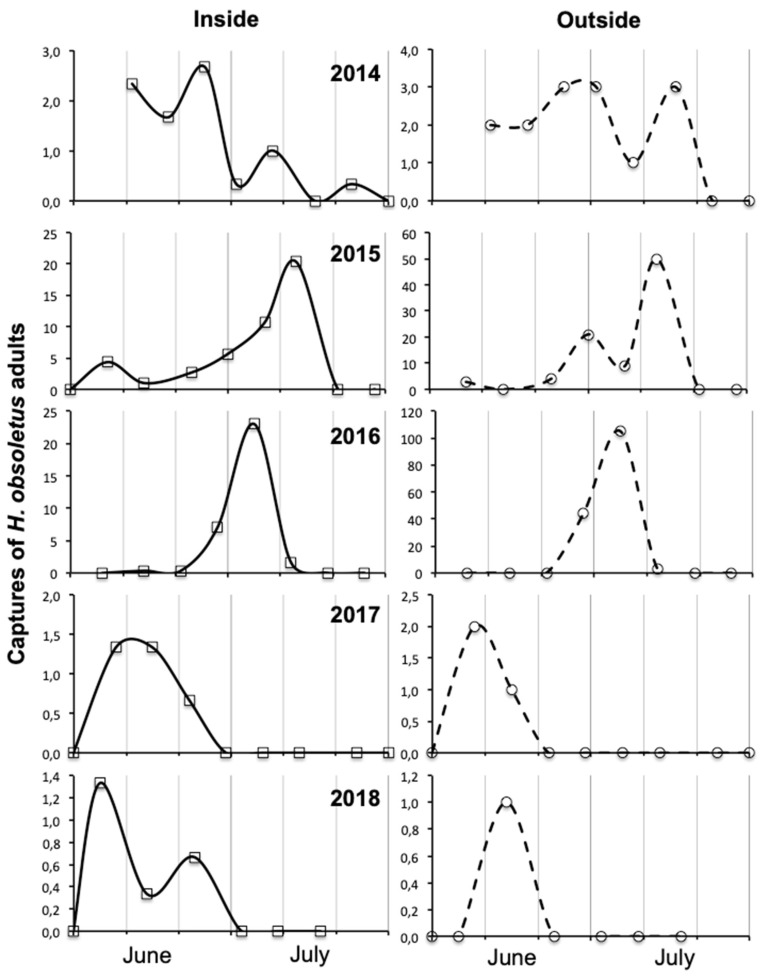
*Hyalesthes obsoletus* captured on yellow sticky traps during 2014–2018 both inside and outside vineyard B. A value of outside captures refers to the sum of seven traps and that of inside to the sum of the average of each of seven series.

In both vineyards, captures were significantly lower in 2017 and 2018 than in the previous three years, showing a significant severe decline in 2017, the effect of which also persisted in the following year (Figure 3 and Figure 4). In both vineyards, captures were higher from late June than in the previous period (Figure 3 and Figure 4). Only in vineyard A, captures were significantly higher outside than inside the vineyard (Figure 3 and Figure 4); in vineyard B, the differences were on average not in contradiction with those of vineyard A, but did not reach the level of statistical significance because captures were higher outside the vineyard in only two out of five years.

Some interactions were also significant (Table 1 and Table 2). The interaction year × period was significant in both vineyard habitats and is explained by the fact that only in 2014–2016 captures were much higher in July than in June (Figure 3 and Figure 4). The interaction year × position was significant only in vineyard A because only in 2014–2016 the captures were much higher along the ditch than inside the vineyard (Figure 3 and Figure 4); however, the data relating to vineyard B, although the interaction did not reach the level of statistical significance, were not in contradiction with those of vineyard A because in two out of five years the captures were much higher outside the vineyard. The interaction period × position was significant in both vineyard habitats because captures were higher in July than in June, more along the ditch than inside the vineyard (Figure 3 and Figure 4).

### 3.4. Bois Noir Incidence and Its Relationship with Adult Abundance

**Figure 5:**
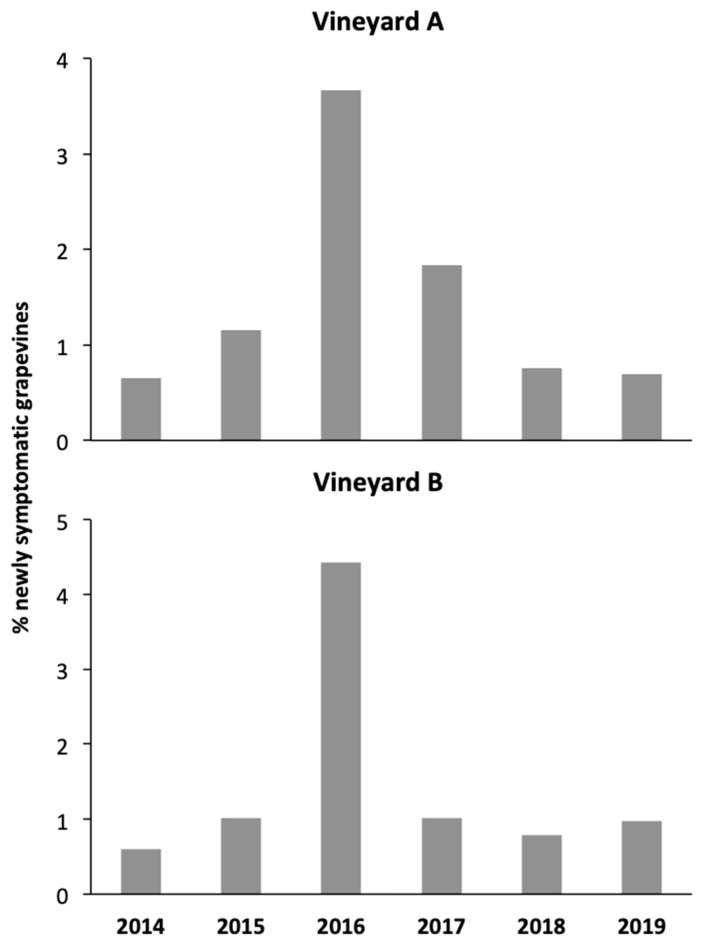
Newly grapevine yellows (GY) symptomatic grapevines recorded in vineyards A and B over the monitoring years.

In vineyard A, the percentage of newly symptomatic grapevines increased rapidly from 2014 to 2016 and then gradually decreased over the following three years (Figure 5). In vineyard B, a similar trend was observed but a more marked decrease occurred between 2016 and 2017 (Figure 5).

In vineyard A, total *H. obsoletus* captures inside the vineyard was not significantly correlated either with the percentage of newly symptomatic grapevines in the same year or with that in the next year (Table 3). In vineyard B, total *H. obsoletus* captures inside the vineyard was significantly correlated with the percentage of newly symptomatic grapevines in the next year. The trend of regressions related to vineyard A, although not significant, was not in contradiction with that of vineyard B.

In vineyard A, until 2016 only the “*Ca*. P. solani” *tuf*-type a was recorded (Table 4). From 2017 a progressive increase of “*Ca*. P. solani” *tuf*-type b was observed, and in the two years 2018–2019 this phytoplasma type was on average prevalent. In vineyard B, “*Ca*. P. solani” *tuf*-type a was always significantly prevalent (Table 4), but a relatively higher incidence of “*Ca*. P. solani” *tuf*-type b was observed on grapevines newly symptomatic in 2017.

## 4. Discussion

### 4.1. Phenology of Adults on Field Bindweed and Stinging Nettle

The emergence of *H. obsoletus* adults started earlier in the season on field bindweed than on stinging nettle, in agreement with the other studies on planthopper phenology on these two host plants [18,26,31,32]. In our study areas, the earlier emergence of adults on field bindweed compared to stinging nettle was due to the fact that nymphs overwinter as third instars on the former plant and as second instars on the latter [19].

Where both field bindweed and stinging nettle were present, the dynamics of *H. obsoletus* captures were bimodal, confirming that the adult emergence on stinging nettle is clearly shifted forward compared to that on field bindweed. These differences in adult phenology recorded in the study areas by sweep net sampling are substantially in agreement with those observed in another grape-growing area of north-eastern Italy using yellow sticky traps [31].

Different adult phenology implies differences in the period in which the phytoplasma causal agent of BN can be inoculated into grapevines. Since the newly emerging adults are already able to transmit phytoplasma [7], the inoculation period begins two weeks earlier for *H. obsoletus* from field bindweed. This can influence the efficacy of control measures applied in vineyards against the adults that colonize grapevines. In northern Italy vineyards, insecticides applied onto the grapevine canopy against other pests influenced neither the disease incidence nor *H. obsoletus* presence [17,46,47,48,49]. This occurrence was explained by the fact that nymphs are soil-dwelling and adults often colonize vineyards from outside [14,17,26,50]. In addition, this study allows us to shed light on the fact that insecticide application at the end of June against *S. titanus* is not timed to control either the *H. obsoletus* adults emerging from field bindweed that peak in mid-June, or those emerging from stinging nettle that peaked in mid-July. Additionally, insecticides applied in August cannot affect *H. obsoletus* populations because only a few adults are still present. Strategies to control this planthopper focus on the nymphs on the roots of their host plants [24,51]. The unavailability of the original host plants and the inability of adults to adapt to a food source different from that on which nymphs developed would drive the vectors to disperse, favoring vineyard colonization [52]. This can be taken into consideration to design a rational BN control strategy.

### 4.2. Survival and Adaptation of Adults on Field Bindweed and Stinging Nettle

Adults of *H. obsoletus* originating from stinging nettle survived better on stinging nettle than on field bindweed and vice versa for adults from bindweed. These data confirm the results of previous studies [35,36,37] and support the hypothesis of cryptic speciation [38,39].

The differences in survival between the two plants were higher for the *H. obsoletus* populations from stinging nettle than from field bindweed, in agreement with previous studies [36,37]. This occurrence is due to the higher survival of adults from stinging nettle than those from field bindweed regarding host plant of origin. The trends in *H. obsoletus* captures recorded in vineyard habitats of northern Italy (see this study and [31]) seem to confirm this occurrence, the flight period on field bindweed being both shorter and concentrated around the peak (for example, after 21 days from the starting of captures, the cumulative percentages of total captures were 64% and 29% for field bindweed and stinging nettle, respectively). Therefore, a longer inoculation period for vector population transmitting “*Ca*. P. solani” *tuf*-type a than those transmitting *tuf*-type b can be supposed. However, since in this study *H. obsoletus* adults were collected on the same date from both the herbaceous plants, the lower survival of adults from field bindweed could be because they were on average older, being that their emergence started before those from stinging nettles.

### 4.3. Influence of Year on Amount, Spatial Distribution and Phenology of Adult Captures

In vineyards A and B, in the years when adult populations of *H. obsoletus* were higher (i.e., 2014–2016), differences in the phenology, dynamics and amounts of captures were observed between outside (i.e., along ditches) and inside vineyards. Along the ditches with stinging nettle the captures of *H. obsoletus* were much higher in July than in June, whereas inside the vineyard with field bindweed in inter-rows captures were often comparable in the two months, in agreement with the data recorded in another grape-growing areas of north-eastern Italy (see [31] and Figure 1 in this paper).

**Figure 6:**
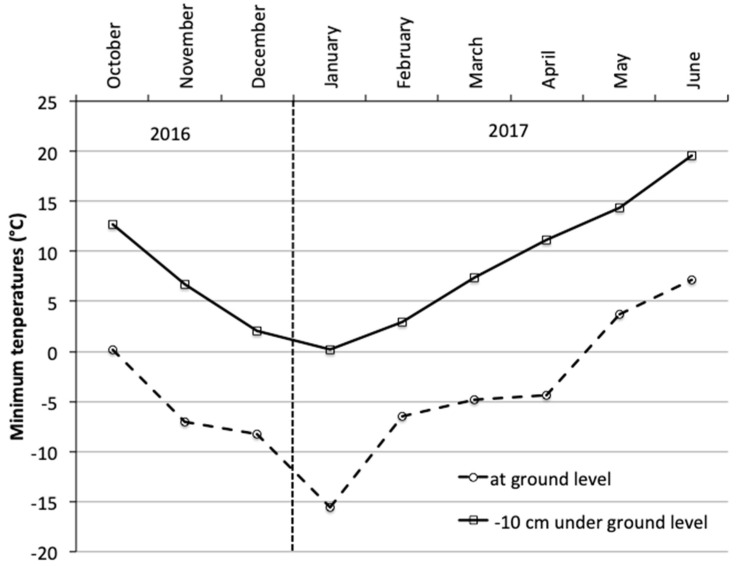
Minimum temperatures recorded during development of *H. obsoletus* nymphs on the roots of herbaceous host plants. In the winter period the nymphs live 20–25 cm underground, but in spring they move to ground level.

In both vineyards, the drop in captures from 2016 to 2017 must be traced back to the meteorological mortality factor as the same phenomenon occurred in both vineyards and both inside and outside. In the literature it is reported that *H. obsoletus* is affected by high average annual precipitation and lowest minimum temperatures during the cold season [53,54], but this is not the case for 2017. From a careful analysis of minimum temperatures from autumn 2016 to spring 2017 (Table S1 and Figure 6; data OSMER Friuli Venezia Giulia), the event that was more likely to be the cause of the strong mortality in insect populations appears to be the late frost that occurred in the early morning of April 21, 2017. In fact, if minimum temperatures at 10 cm of depth never fell below 0 °C, it is unthinkable that they would have killed the nymphs of *H. obsoletus* who overwinter at 20–25 cm of depth [15]. In the area of the study vineyards, the temperatures at ground level remained below 0 °C for 4 h with a minimum of −4.4 °C and severe frost damage was observed on the grapevines in the subsequent days [55]. It is known that this planthopper finds shelter from the winter rigors by going deeper in the ground and that in the spring it moves to the more superficial layers [26]. On 21 April, when the temperature at ground level had dropped to −4.4 °C, the temperature measured in the soil at a depth of 10 cm reached a minimum of 11.3 °C. It is clear that if the frost had occurred when the nymphs were still protected in the ground, they would not have been injured. This study therefore highlighted another factor that may affect the vector’s population density.

In the three-year period 2014–2016 compared to the two-year period 2017–2018 the captures of *H. obsoletus* were significantly higher outside than inside the vineyard and in July than in June because there was a prevalence of populations from stinging nettle. In the second period, there was a higher importance of planthopper populations that emerged from field bindweed inside the vineyards in June. The change in relative importance of *H. obsoletus* populations from stinging nettle to field bindweed could be explained by a greater susceptibility to low temperatures in spring of the nymphs developing on stinging nettle, because at the frost date they were on average smaller, as a consequence of the younger overwintering instars [19]. Since in vineyard B the captures along the ditch with stinging nettle were low not only in 2017 but also in 2018, it can be deduced that the populations emerged from field bindweed did not colonize stinging nettle reinforcing the idea of the cryptic speciation.

### 4.4. Relationship between Adult Populations and Newly Symptomatic Grapevines

Many studies have highlighted a relationship between the abundance of *H. obsoletus* and its herbaceous host plants and the incidence of BN in vineyards [17,28,29,53,56,57,58,59], but only a few have attempted to relate a measured density of the vector with the increase of newly symptomatic grapevines [17,58]. In the case of annual herbaceous plants, stolbur phytoplasma incidence is directly associated with *H. obsoletus* density in the same year [60], whereas in the case of tree plants the symptoms usually occur in the following years’ inoculation [61,62]. However, on one-year grapevines, symptoms of BN can appear for the first time in the late season of the planting year, i.e., the same year in which they are exposed to natural infections for the first time [63]. In the study of Mori et al. [17], where the data set (i.e., *H. obsoletus* density/newly symptomatic grapevines) referred to different vineyards, the newly symptomatic grapevines were not associated with either density of the vector inside the vineyard in the current year or that of the previous year.

In vineyard B of this study, the newly symptomatic grapevines were significantly related to captures of *H. obsoletus* recorded in the previous year, which indirectly confirms that symptoms often occur the year subsequent to inoculation. However, the data concerning vineyard A did not confirm this.

In vineyard A, a reduction in newly symptomatic grapevines occurred the year after the collapse of *H. obsoletus* populations, whereas that occurred in the same year in vineyard B. Since the drop in *H. obsoletus* populations can be reasonably associated with the late frost that occurred in spring 2017, a relationship between late frosts and dynamics of newly symptomatic grapevines in the vineyard can be supposed. For this purpose, based on data collected on BN-infected vineyards in northeastern Italy during 1987–2000 [64], the net decrease in percentage of newly symptomatic grapevines that occurred in 1997 was observed in coincidence with a late frost (data OSMER Friuli Venezia Giulia). Therefore, it can be supposed that the dynamics of BN in the vineyard can be influenced by late frosts that periodically cause the collapse of *H. obsoletus* populations.

### 4.5. Relationship between Adult Population and “Ca. P. solani” Tuf-Type

In the habitat of vineyard A, in the years when captures were high along the ditch with stinging nettle (2014–2016) and the highest incidence of newly symptomatic grapevines occurred, only the “*Ca*. P. solani” *tuf*-type a, i.e., the type that associated with stinging nettle, was recorded. In the years after the drop in *H. obsoletus* populations along the ditch (2017–2018), captures inside the vineyard decreased mostly in July in comparison with the previous years and on average a prevalence of *tuf*-type b, i.e., the type that associated with field bindweed, was recorded. This occurrence was also favored by the fact that from 2016 a large surface of inter-rows was covered by field bindweed as a consequence of inter-row tillage.

In the habitat of vineyard B, despite the absence of captures in July 2017 and 2018 both inside and outside the vineyard, the *tuf*-type a remained the prevalent type.

## 5. Conclusions

The populations of *H. obsoletus* adults developing on *C. arvensis* and *U. dioica* showed two important differences: (i) adult phenology was earlier on field bindweed than on stinging nettle, (ii) adult emergence inside vineyards was rare when the prevalent herbaceous host was *U. dioica*, whereas it was important when *C. arvensis* was prevalent. The differences in adult phenology cannot be attributed to a faster developmental time of nymphs on field bindweed because their older overwintering nymphal instars can be explained by an earlier oviposition period by adults emerged from this host plant. Moreover, the fact that differences in adult phenology are consistent year after year suggest that adults emerged from *C. arvensis* do not lay eggs on *U. dioica* and vice versa, thereby reinforcing the idea of the cryptic speciation. The differences in phenology and spatial distribution of the vector involve different periods of inoculation into grapevines of the two “*Ca*. P. solani” *tuf*-types and a different importance of vector population colonizing vineyards from outside, and as a consequence the BN control strategies must be differentiated.

The populations of *H. obsoletus* varied widely from year to year and late frosts in spring could be an important abiotic mortality factor. Evident decreases in newly symptomatic grapevines in the same or the following year could be explained by drops in vector populations occurring as a consequence of a late frost in spring.

The change in the proportion of *H. obsoletus* from field bindweed or stinging nettle was associated with a different incidence of the two *tuf*-types in newly symptomatic grapevines.

## Figures and Tables

**Table 1:** Captures of *Hyalesthes obsoletus* recorded from 2014 to 2018 in vineyard A habitat.

Source of Variation	Levels	Mean ± SE	F	Degree of Freedom	*p*
Year	2014	13.07 ± 6.10 c	30.36	4, 118	<0.0001
	2015	10.76 ± 5.15 c			
	2016	6.13 ± 2.49 b			
	2017	0.196 ± 0.085 a			
	2018	0.241 ± 0.180 a			
Period	1–25 June	0.750 ± 0.147	61.15	1, 118	<0.0001
	26 June to 20 July	11.41 ± 3.31			
Position	Outside	11.09 ± 3.32	25.38	1, 118	<0.0001
	Inside	1.08 ± 0.191			
Replicate			3.30	6, 118	0.005
Year × period			9.26	4, 118	<0.0001
Year × position			2.86	4, 118	0.026
Period × position			45.22	1, 118	<0.0001

F and *P* are values of ANOVA. Different small letters among the captures recorded in the five years indicate significant differences according to Tukey’s test (α = 0.01).

**Table 2:** Captures of *Hyalesthes obsoletus* recorded from 2014 to 2018 in vineyard B habitat.

Source of Variation	Levels	Mean ± SE	F	Degree of Freedom	*p*
Year	2014	0.961 ± 0.278 bc	18.67	4, 118	<0.0001
	2015	4.07 ± 1.20 d			
	2016	4.45 ± 2.26 d			
	2017	0.214 ± 0.084 ab			
	2018	0.083 ± 0.033 a			
Period	1–25 June	0.610 ± 0.123	13.31	1, 118	<0.0001
	26 June to 20 July	3.303 ± 1.036			
Position	Outside	3.033 ± 1.035	2.98	1, 118	0.087
	Inside	0.880 ± 0.191			
Replicate			2.10	6, 118	0.059
Year × period			17.34	4, 118	<0.0001
Year × position			2.02	4, 118	0.096
Period × position			12.54	1, 118	0.001

F and *P* are values of ANOVA. Different small letters among the captures recorded in the five years indicate significant differences according to Tukey’s test (α = 0.01).

**Table 3:** Regressions between captures of *Hyalesthes obsoletus* on herbaceous vegetation inside the vineyards (2014–2018) and percentage of newly GY symptomatic grapevines in the same (2014–2018) and in the next year (2015–2019).

% Newly Symptomatic Grapevines	Vineyard	Equation	*P*	R^2^
In the same year	A	Y = −0.009X + 2.17	0.48	0.18
	B	Y = 0.016X + 1.01	0.60	0.10
In the next year	A	Y = 0.014X + 0.71	0.19	0.49
	B	Y = 0.047X + 0.15	0.032	0.83

**Table 4:** **“***Ca*. P. solani” *tuf*-type identified on GY symptomatic grapevines in vineyards A and B.

Vineyard/Year	Total Newly Symptomatic Grapevines	Newly Symptomatic Grapevines Analysed	*Tuf*-Type a		*Tuf*-Type b		*P* at Binomial Test
	N.	N.	N.	%	N.	%	
Vineyard A							
2016	56	21	21	100	0	0.0	>0.0001
2017	20	8	5	62.5	3	37.5	NS
2018–2019 ^1^	15	6	2	33.3	5	83.3	NS
Vineyard B							
2015–2016	90	20	19	95.0	1	5.0	>0.0001
2017	16	13	10	76.9	3	23.1	0.046
2018–2019	27	14	13	92.9	1	7.1	0.0009

^1^ One grapevine had a mixed infection with both *tuf*-types; thus, the total number of grapevines with the two *tuf*-types was higher than the number of analyzed grapevines and the sum of percentages was higher than 100%.

## Supplementary Materials

The following are available online at https://www.mdpi.com/2075-4450/11/9/606/s1. Table S1: Temperature data from the weather station of Gradisca d’Isonzo (Gorizia district, Friuli Venezia Giulia region, Italy) (https://www.osmer.fvg.it).
